# Dataset of spectroscopic, crystallography and DFT of novel 1,2-bis[*N,N’*-6-(4-pyridylmethylamido)pyridyl-2-carboxyamido]butane

**DOI:** 10.1016/j.dib.2023.109635

**Published:** 2023-10-04

**Authors:** M.A. Kadir, N.S.H. Haris, M.S.M. Yusof, K. Kassim, Fazira Ilyana Abdul Razak

**Affiliations:** a Faculty of Science and Marine Environment, Universiti Malaysia Terengganu, 21030 Kuala Nerus, Terengganu, Malaysia; b Centre of Chemical Synthesis and Polymer Technology (CCSPT), Institute of Science (IOS), Universiti Teknologi Mara, Shah Alam, 40450, Shah Alam Selangor Malaysia; c Department of Chemistry, Faculty of Science, Universiti Teknologi Malaysia, 81310, UTM Johor Bahru, Johor, Malaysia

**Keywords:** FTIR, NOESY NMR, Crystal, Synthesis, Structure

## Abstract

This paper provided the dataset obtained from spectroscopic, crystallography and DFT of a new compound namely 1,2-bis[*N,N’*-6-(4-pyridylmethylamido)pyridyl-2-carboxyamido]butane. This compound is prepared from the reaction between *N*-6-[(4-pyridylmethylamino)carbonyl]-pyridine-2-carboxylic acid methyl ester with butane-1,4-diamine. The preparation of this compound is modified from the method described in our article [1]. In this work, we present data characterization of 1,2-bis[*N,N’*-6-(4-pyridylmethylamido)pyridyl-2-carboxyamido]butane from Fourier Transform Infrared (FTIR), ^1^H Nuclear Magnetic Resonance (^1^ H NMR), NOESY NMR, ^13^C Nuclear Magnetic Resonance (^13^C NMR), and elemental analysis (CHNS). The structure of this molecule is also analysed by X-ray crystallography and DFT studies. A single-crystal X-ray diffraction investigation was carried out by using Bruker SMART Apex II Duo CCD area-detector diffractometers with MoKα radiation (wavelength of λ = 0.71073 Å). The optimized energy was indicated with GaussView 5.0 and Gaussian 16 software package programme.

Specifications Table

**Table:** 

Subject	Chemistry
Specific subject area	Synthetic chemistry, computational chemistry, crystallography, spectroscopy, organic chemistry, inorganic chemistry
Data format	Raw, Analyzed, Filtered, JPEG, Tiff (Raw)
Type of data	FTIR spectrum, NMR spectrum, UV spectrum, Table, Image.
Data collection	The spectra were acquired in the 4000-400 cm^−1^ range using an ATR-FTIR spectrophotometer. The Perkin Elmer 100 model was utilized for FTIR analysis. Nuclear Magnetic Resonance spectra were recorded using a Bruker Advance II 400 spectrometer. UV-Vis spectrum was obtained using the Shimadzu UV-1800 Spectrophotometer. CHNS Analyzer Flash EA 1112 was employed to record CHN results. Optimized structure of 1,2-bis[*N,N’*-6-(4-pyridylmethylamido)pyridyl-2- carboxyamido]butane was accomplished using GaussView 5.0 and Gaussian 16 software package programme [2]. X-ray diffraction data were obtained utilizing Mo-Kα radiation (wavelength λ = 0.71073 Å). The data collection was performed at 150(2) K using an Oxford Diffraction X-Calibur single-crystal X-ray diffractometer. Absorption correction was applied to all datasets through a multi-scan approach. The structures were initially solved using SHELXS-97 and subsequently refined using full-matrix least-squares on F2 with SHELXL-97 [3] interfaced through the program X-Seed [4]. The molecular graphics were drawn using SHELXTL [5]. The isotropic displacement parameters are set to 1.2(C) times the equivalent isotropic U values of the parent carbon atoms. All the hydrogen atoms were physically positioned (C—H = 0.93) and refined using the riding model U_iso_(H) = 1.2 U_eq_(C). Additionally, a different Fourier map was utilized to locate and refine the N-bound hydrogen atoms (N—H = 0.86).
Data source location	Universiti Malaysia Terengganu Universiti Sains Malaysia Universiti Teknologi Mara Universiti Teknologi Malaysia
Data accessibility	All data is provided in this article. The crystal structure has been deposited as following details: Repository name: Cambridge Crystallographic Data Centre Data identification number: CCDC 2277065 url: http://www.ccdc.cam.ac.uk/services/structures?access=referee&searchdepnums=2277065&searchauthor=Kadir
Related research article	Haris NSH, Mansor N, Yusof MSM, Sumby CJ, Kadir MA (2021) Investigating the potential of flexible and pre-organized tetraamide ligands to encapsulate anions in one-dimensional coordination polymers: synthesis, spectroscopic studies and crystal structures. Crystals, 11: 77. https://doi.org/10.3390/cryst11010077.

## 1 Value of the Data

• The information derived from the integration of FTIR, NMR, and UV-Vis spectroscopic techniques is valuable for both characterizing and confirming the structures of novel compounds.
• The knowledge obtained from theoretical and crystallography data can benefit researchers from physical chemistry and crystallographers in understanding the molecule stability in the solid state.
• The data provided in this article can be reused by other researcher in their effort to produce new compounds potentially suitable for anion separation materials.

## 2 Data Description

A new compound namely 1,2-bis[*N,N’*-6-(4-pyridylmethylamido)pyridyl-2-carboxyamido]butane has been successfully synthesized from reaction between *N*-6-[(4-pyridylmethylamino)carbonyl]-pyridine- 2-carboxylic acid methyl ester with butane-1,4-diamine in toluene [1]. Characterization of 1,2-bis[*N,N’*-6-(4-pyridylmethylamido)pyridyl-2-carboxyamido]butane was achieved through a combination of spectroscopic techniques, namely Fourier Transform Infrared (FTIR), ^1^H Nuclear Magnetic Resonance (^1^ H NMR), NOESY NMR, ^13^C Nuclear Magnetic Resonance (^13^C NMR), and elemental analysis (CHNS).

**Fig. 1:**
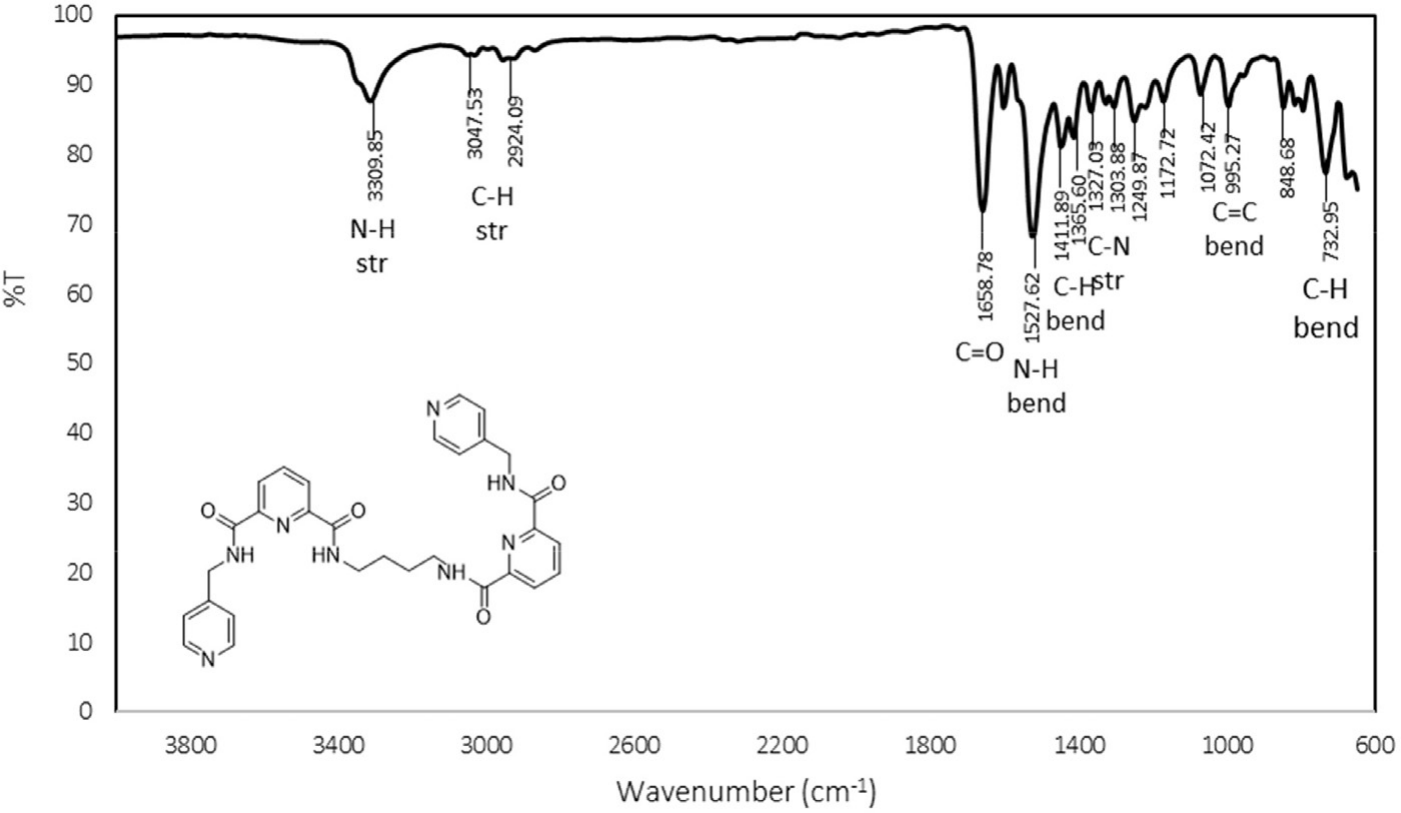
FTIR spectrum of 1,2-bis[*N,N’*-6-(4-pyridylmethylamido)pyridyl-2-carboxyamido]butane.

**Fig. 2:**
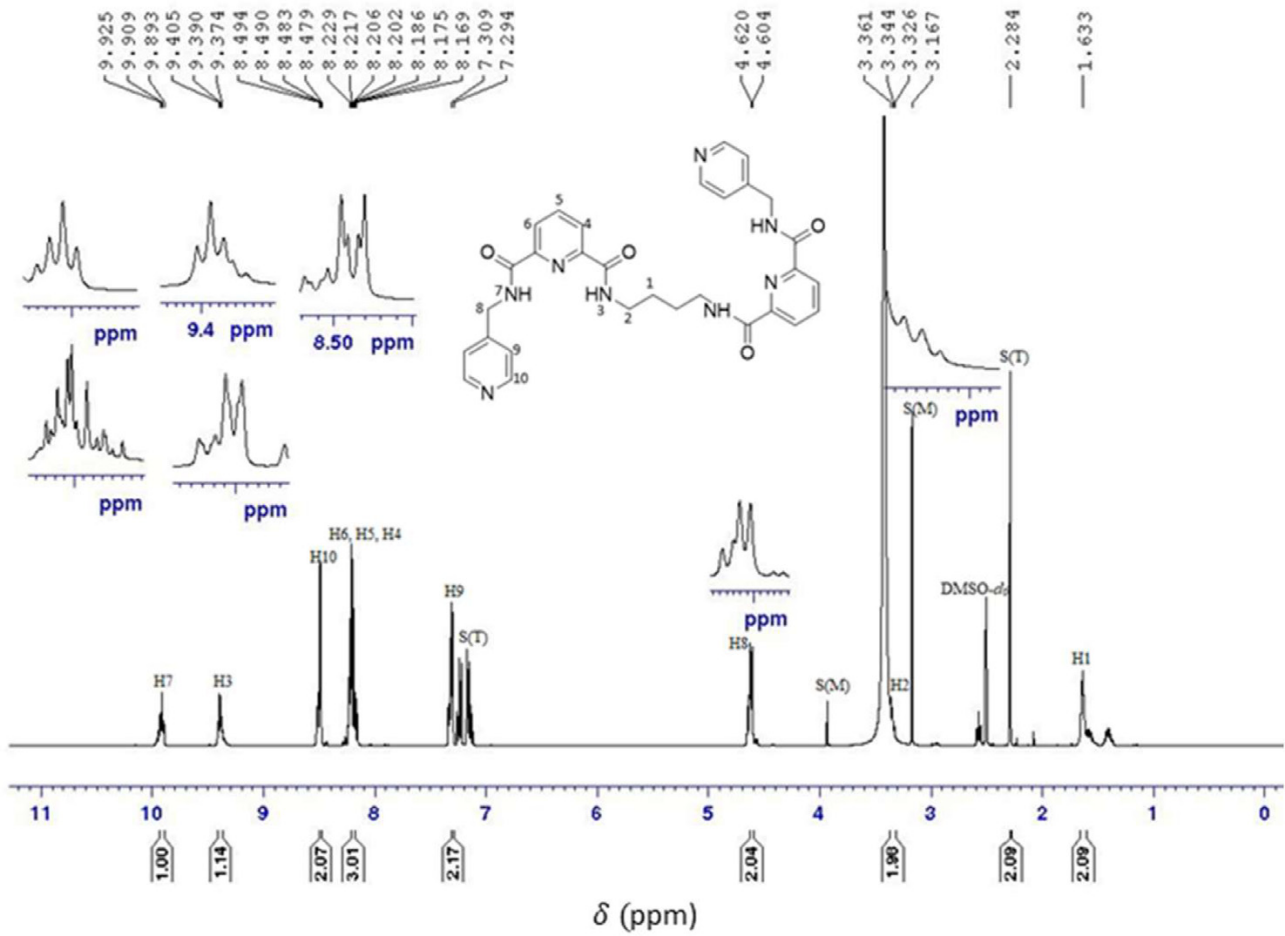
^1^H NMR spectrum of 1,2-bis[*N,N’*-6-(4-pyridylmethylamido)pyridyl-2-carboxyamido]butane.

**Fig. 3:**
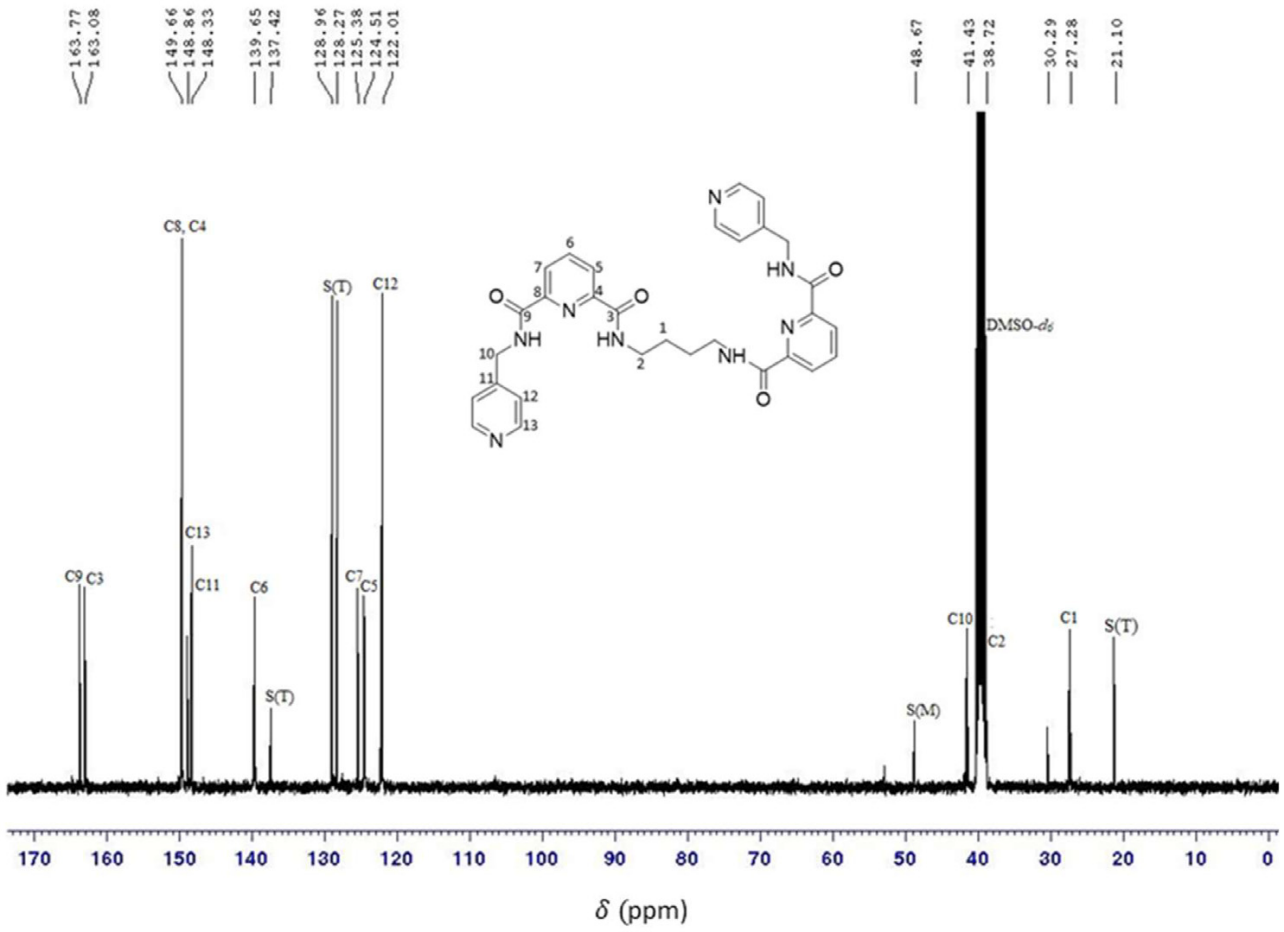
^13^C NMR spectrum of 1,2-bis[*N,N’*-6-(4-pyridylmethylamido)pyridyl-2-carboxyamido]butane.

**Fig. 4:**
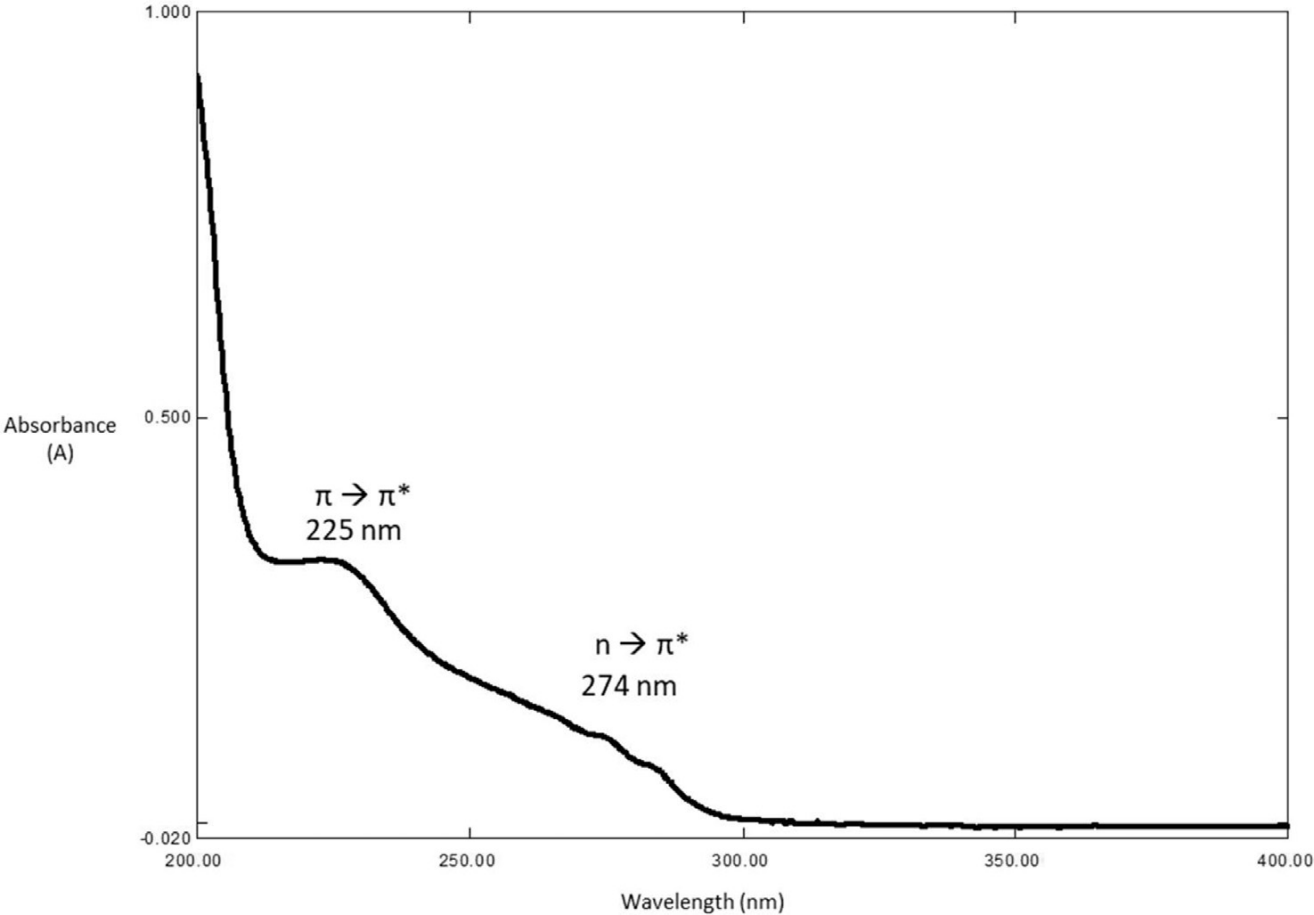
UV-Vis spectrum of 1,2-bis[*N,N’*-6-(4-pyridylmethylamido)pyridyl-2-carboxyamido]butane.

**Fig. 5:**
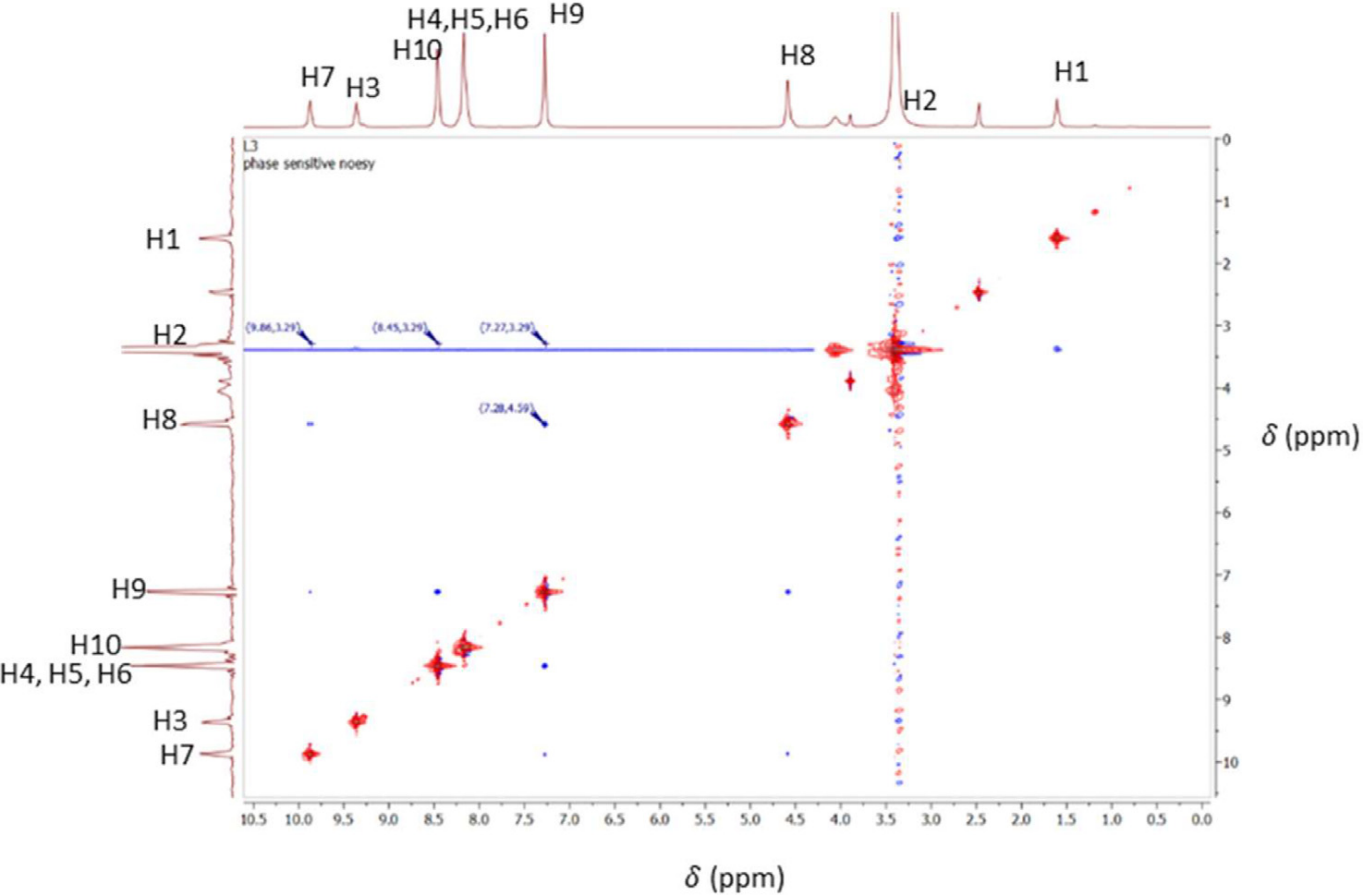
NOESY NMR spectrum of 1,2-bis[*N,N’*-6-(4-pyridylmethylamido)pyridyl-2-carboxyamido]butane.

The FTIR spectra contain a number of significant peaks, including the ν(N—H)_str_, ν(C—H)_str_, ν(C=O), ν(N—H)_bend_, ν(C—H)_bend_, ν(C—H)_str_, and ν(C=C)_bend_ peaks, which were found at 3309 cm^−1^, 3047 cm^−1^, 1658 cm^−1^, 1527 cm^−1^, 1411 cm^−1^, 1327 cm^−1^, and 995 cm^−1^, respectively. In the ^1^H NMR spectrum, the protons of the alkyl, pyridine, and amide groups were detected at 1.63–4.62 ppm, 7.30–8.49 ppm, and 9.39–9.90 ppm, respectively. Meanwhile, in the ^13^C NMR, the resonances for the carbons of alkyl, pyridine, and carbonyl groups were found in the range of 27.28–41.43 ppm, 122.01–149.66 ppm, and 163.08–163.94 ppm, respectively. The electronic transitions revealed two chromophore absorption peaks, pyridine (C=C) and carbonyl (C=O), which have λ_max_ absorption bands at 225 nm and 274 nm, respectively. These transitions correspond to π-π* and n-π* transitions. In the NOESY NMR spectrum, two distinct arrangements were observed, manifesting as patterns centred around the proton in the alkyl group (H2) at 3.34 ppm. Initially, the chemical shift of H2 in the spacer region at 3.34 ppm exhibited a cross-peak correlation with H7, where strong intramolecular correlations can be observed. These patterns also demonstrated cross-peak correlations with signals from the amide group (H2) and the pyridine group, specifically H9 (7.30 ppm) and H10 (8.49 ppm), respectively. The spectral information is illustrated in Fig. 1, Fig. 2, Fig. 3, Fig. 4, Fig. 5, respectively. Meanwhile, the summarized data were tabulated in Table 1, Table 2, Table 3, Table 4, respectively.

**Table 1:** FTIR data for 1,2-bis[*N,N’*-6-(4-pyridylmethylamido)pyridyl-2-carboxyamido]butane.

Vibrational Modes	Wavenumber (cm^−1^)
N—H_str_	3309
C—H_str_	3047
CO	1658
N—H_bend_	1527
C—H_bend_	1411
C—N	1327
CC	995

**Table 2:** ^1^H NMR data for 1,2-bis[*N,N’*-6-(4-pyridylmethylamido)pyridyl-2-carboxyamido]butane.

Assignment	Moiety	Chemical shift (δ_H,_ ppm)
H1	q, 2H, *CH_2_*CH_2_NH	1.63
H2	q, *J_HH_* = 6.8 Hz, 2H, CH_2_*CH_2_*NH	3.34
H8	d, *J_HH_* = 6.4 Hz, 2H, py*CH_2_*NH	4.62
H9	d, *J_HH_* = 6 Hz, 2H, C_5_H_4_	7.30
H4, H5, H6	m, 3H, C_5_H_3_	8.20
H10	d, *J_HH_* = 4.4 Hz, *J_HH_* = 1.6 Hz, 2H, C_5_H_4_	8.49
H3	t, *J_HH_* = 6 Hz, 1H, CH_2_CH_2_*NH*	9.39
H7	t, *J_HH_* = 6.4 Hz, 1H, pyCH_2_*NH*	9.90

**Table 3:** ^13^C NMR for 1,2-bis[*N,N’*-6-(4-pyridylmethylamido)pyridyl-2-carboxyamido]butane.

Assignment	Moiety	Chemical shift(δ_H,_ ppm)
C1	*CH_2_*CH_2_NH	27.28
C2	CH_2_*CH_2_*NH	38.72
C10	py*CH_2_*NH	41.43
C12	C=C	122.01
C5	C=C	124.51
C7	C=C	125.38
C6	C=C	139.65
C11	C=C	148.33
C13	C=C	148.86
C4, C8	C=C	149.66
C3	C=O	163.08
C9	C=O	163.77

**Table 4:** The UV-Vis data for 1,2-bis[*N,N’*-6-(4-pyridylmethylamido)pyridyl-2-carboxyamido]butane.

Chromophores	Transition	λ_max_(nm)
Pyridine	π-π*	225
C=O	n-π*	274

**Fig. 6:**
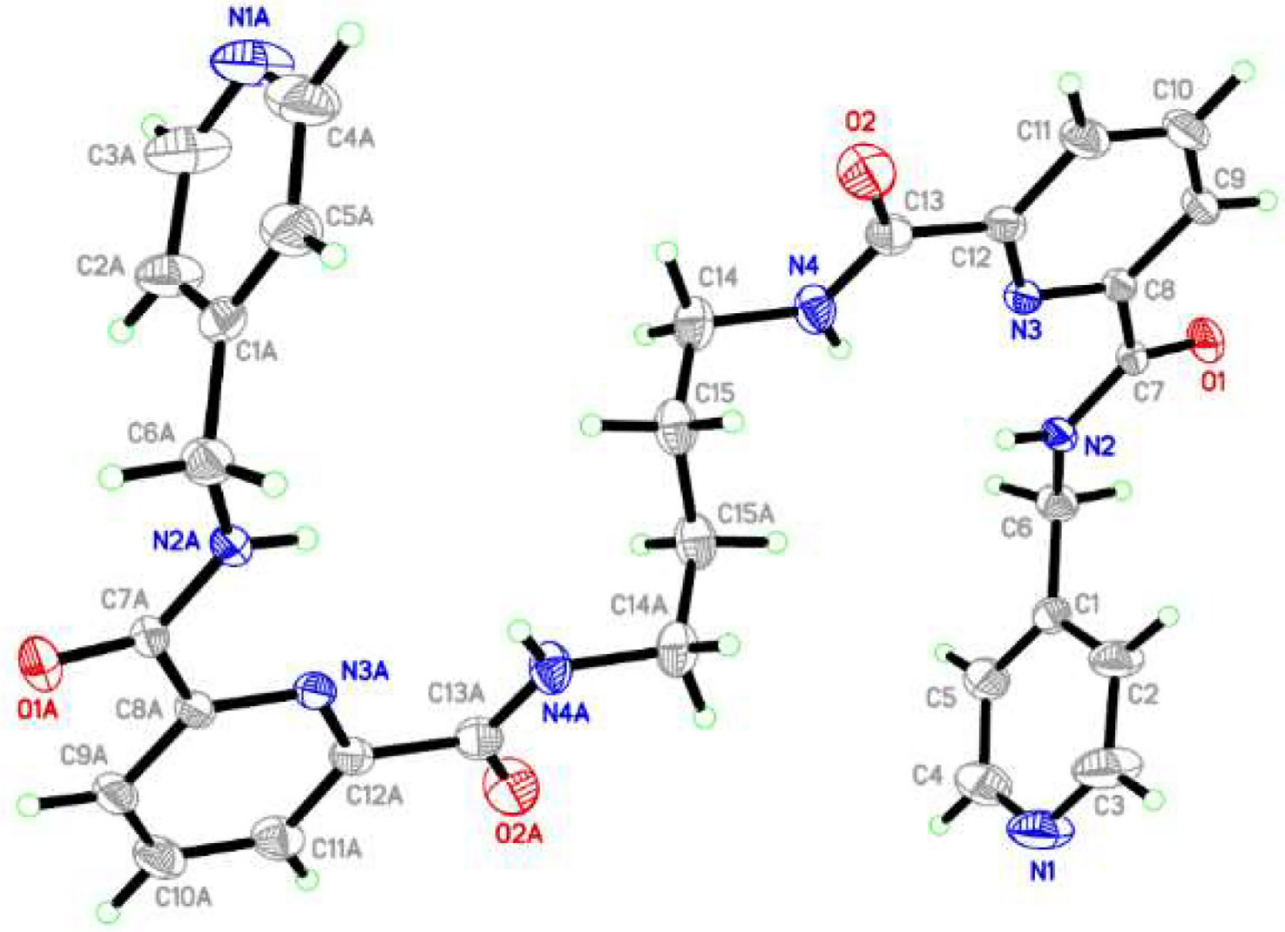
ORTEP structure of compound with ellipsoids shown at the 50 % probability level (C14—N4=1.425 Å, C13—O2=1.195 Å, C7—O1=1.209 Å, N3—C8=1.316 Å, N3—C12= 1.307 Å, C11—C12=1.357 Å, C12—C13=1.465 Å, C14—C15=1.475 Å, N2—C7=1.297 Å).

**Fig. 7:**
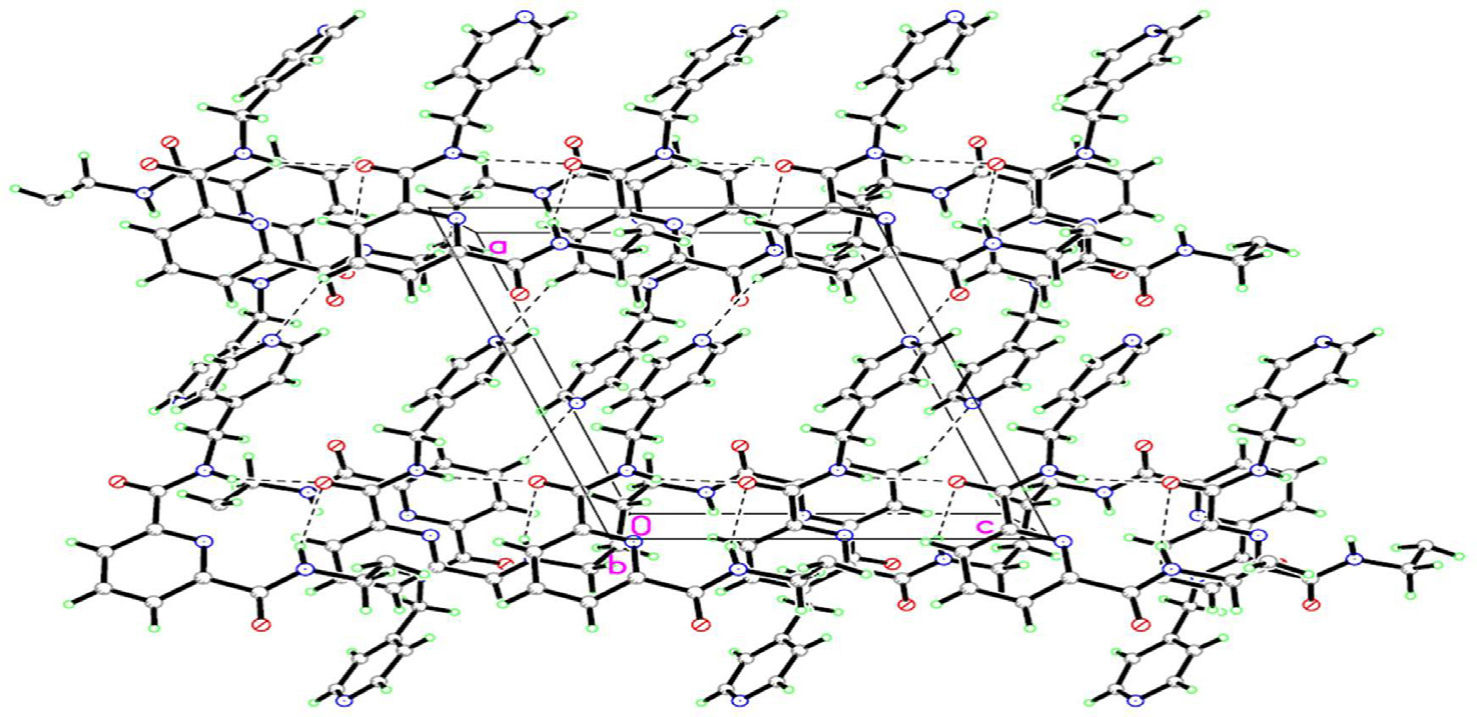
Packing structure when viewed from right side the *c*-axis.

**Fig. 8:**
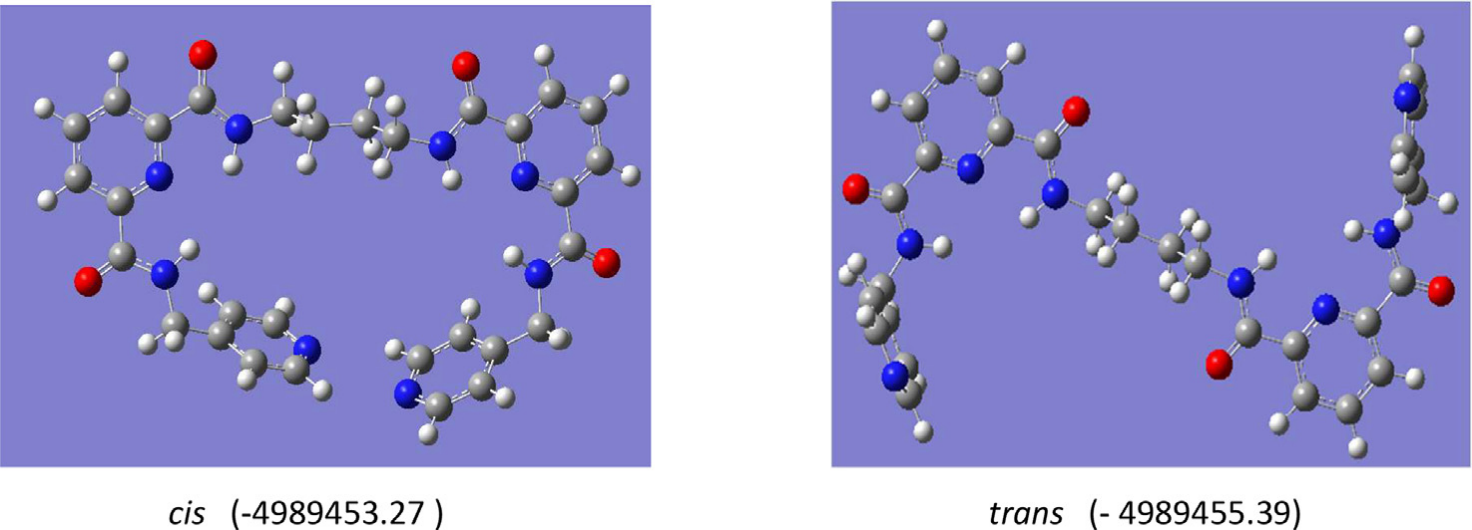
Optimization energies data for 1,2-bis[*N,N’*-6-(4-pyridylmethylamido)pyridyl-2-carboxyamido]butane.

Compound 1,2-bis[*N,N’*-6-(4-pyridylmethylamido)pyridyl-2-carboxyamido]butane was crystallized in a monoclinic crystal system, adopting the *P21/c* space group. The crystal structure revealed that this compound adopted a *trans* conformation, attributed by rotation at butyl spacer (C14A—C15A—C15—C14) across with torsion angles 111°. The 2,6-pyridine dicarboxamide moieties and pyridyl of the pendant arms in the ligand were oriented in the opposite direction, as depicted in Fig. 6. In the crystal packing, the molecules were connected by N—H⋯O hydrogen bonds (Fig. 7). Meanwhile, Tables 5 and 6 contain a tabulation of the datasets from crystal data. DFT studies supported the *trans* conformation and the optimization energy is −4989455.39 kJ/mol (Fig. 8).

**Table 5:** Hydrogen-bond geometry (Å, ${}^{\circ }$).

D—H⋯A	D—H	H⋯A	D⋯A	D—H⋯A
N(2)—H(1)⋯N3	0.85	2.25	2.648(3)	108.7
N(2)—H(1)⋯O(1)	0.85	2.13	2.865(3)	144.3
N(4)—H(1)⋯N(3)	0.81	2.25	2.634(3)	110
N(4)—H(1)⋯O(1)	0.81	2.46	3.216(3)	157
C(6)—H(6)⋯O(1)	0.97	2.39	2.768(3)	103
C(10)—H(10)⋯N(1)	0.93	2.48	3.284(5)	145

Symmetry codes: (i) x,1/2-y,1/2+z (ii) -1+x,y,-1+z.

**Table 6:** Crystal data of 1,2-bis[*N,N’*-6-(4-pyridylmethylamido)pyridyl-2-carboxyamido]butane.

Data	1,2-bis[*N,N’*-6-(4-pyridylmethylamido)pyridyl-2-carboxyamido]butane
Empirical formula	C_30_H_30_N_8_O_4_
Formula weight	566.62
Crystal system	Monoclinic
Space group	*P 21/c*
a (Å)	11.434(3)
b (Å)	12.820(4)
c (Å)	9.903(3)
$\alpha ({}^{\underset{‾}{\circ }})$	90
$\beta ({}^{\underset{‾}{\circ }})$	110.905(4)
$\gamma ({}^{\underset{‾}{\circ }})$	90
Volume (Å^3^)	1356.0(6)
Z	2
Density (calculated) (Mg/m^3^)	1.388
Absorption coefficient (mm^−1^)	0.096
F(000)	596
Crystal size (mm^3^)	0.539 × 0.519 × 0.250
Theta range for data (°)	1.907–27.492
Reflections collected	3112
Observed reflections [I>2s(I)]	1590
Data/restraints/parameters	1590/0/198
Goodness-of-fit on F^2^	1.006
R_1_ [I>2s(I)]	0.000
wR_2_ (all data)	0.1331
Largest diff. peak and hole (e.Å^−3^)	0.223 and −0.160

## 3 Experimental Design, Materials and Methods

### 3.1 Synthesis of 1,2-bis[N,N’-6-(4-pyridylmethylamido)pyridyl-2-carboxyamido]butane

*N*-6-[(4-Pyridylmethylamino)carbonyl]-pyridine-2-carboxylic acid methyl ester (1.0107 g, 3.7 mmol) and butane-1,4-diamine (0.19 mL, 1.9 mmol) was suspended in toluene (40 mL) and heated at reflux under inert atmosphere for 55 hours. The reaction was monitored using the thin layer chromatography (TLC) technique until completion. Once the reflux process was completed, the solvent (toluene) was removed using a rotary evaporator (55°C at 70 mbar). The product was obtained as aqueous liquid after removing the solvent. After two days, the aqueous liquid underwent solidification, resulting in the formation of a sticky yellow precipitate as the final product. (1.03 g, 62 %) Mp 122–124 °C. Anal. Calc. for C_30_H_30_N_8_O_4_ (566.61 g/mol): C, 63.59 %; H, 5.34 %; N, 19.78 %. Found: C, 63.46 %; H, 5.44 %; N, 19.05 %.

### 3.2 DFT studies

All calculations were performed by Gaussian 16 using high performance computer (HPC) provided by CICT, Universiti Teknologi Malaysia along with Gauss View 5.0 for visualizations and using the Gaussian16 (G16) program package. Geometries were fully optimized without imposing constraints on bond lengths, bond angles, or dihedral angles. The “OPT” keyword was employed to conduct geometry optimizations using the unrestricted DFT method at the B3LYP/6-311G(d,p) level [2,6]. The basis set 6-311G(d,p) was applied to the C, H, N, and O atoms to optimize the molecular geometry at the B3LYP theoretical level.

## Limitations

Not applicable.

## Ethics Statement

This article does not contain any studies involving human subjects, animal experiments, or any data collected from social media platforms.

## CRediT authorship contribution statement

**M.A. Kadir:** Conceptualization, Writing – original draft, Writing – review & editing. **N.S.H. Haris:** Investigation, Methodology. **M.S.M. Yusof:** Resources. **K. Kassim:** Resources. **Fazira Ilyana Abdul Razak:** Resources, Validation.

## Data Availability

CCDC 2277065 (Original data) (CCDC)

## Appendix Supplementary materials

Image, application 1

Image, application 2
