# Supplementary material for: Dataset of spectroscopic, crystallography and DFT of novel 1,2-bis[N,N’-6-(4-pyridylmethylamido)pyridyl-2-carboxyamido]butane
Source: Data Brief. 2023 Oct 4;51:109635. doi: 10.1016/j.dib.2023.109635 (PMC10570947; doi:10.1016/j.dib.2023.109635)

checkCIF/PLATON report

Structure factors have been supplied for datablock(s) mo_s4b_0m

THIS REPORT IS FOR GUIDANCE ONLY. IF USED AS PART OF A REVIEW PROCEDURE FOR PUBLICATION, IT SHOULD NOT REPLACE THE EXPERTISE OF AN EXPERIENCED CRYSTALLOGRAPHIC REFEREE.

No syntax errors found. CIF dictionary Interpreting this report

Datablock: mo_s4b_0m

```
Bond precision:   C-C = 0.0039 A          Wavelength=0.71073

Cell:              a=11.434(3)      b=12.820(4)          c=9.903(3)
                   alpha=90         beta=110.905(4)      gamma=90
Temperature:       296 K

                 Calculated                  Reported
Volume           1356.1(7)                   1356.0(6)
Space group      P 21/c                      P 21/c
Hall group       -P 2ybc                     -P 2ybc
Moiety formula   C30 H30 N8 O4               C30 H30 N8 O4
Sum formula      C30 H30 N8 O4               C30 H30 N8 O4
Mr               566.62                      566.62
Dx,g cm-3        1.388                       1.388
Z                2                           2
Mu (mm-1)        0.096                       0.096
F000             596.0                       596.0
F000'            596.24
h,k,lmax         14,16,12                    14,16,12
Nref             3112                        3112
Tmin,Tmax        0.950,0.976                 0.723,0.927
Tmin'            0.950

Correction method= # Reported T Limits: Tmin=0.723 Tmax=0.927
AbsCorr = MULTI-SCAN

Data completeness= 1.000           Theta(max)= 27.492

R(reflections)= 0.0577( 1590)                     wR2(reflections)=
                                                  0.1551( 3112)
S = 1.006                  Npar= 198
```

The following ALERTS were generated. Each ALERT has the format
test-name_ALERT_alert-type_alert-level.
Click on the hyperlinks for more details of the test.

Alert level B

```
PLAT230_ALERT_2_B Hirshfeld Test Diff for    C1       --C2       .       10.0 s.u.
```

Alert level C

```
ABSTY02_ALERT_1_C  An _exptl_absorpt_correction_type has been given without
            a literature citation. This should be contained in the
            _exptl_absorpt_process_details field.
            Absorption correction given as multi-scan
PLAT241_ALERT_2_C High   'MainMol' Ueq as Compared to Neighbors of         C3 Check
PLAT242_ALERT_2_C Low    'MainMol' Ueq as Compared to Neighbors of         C1 Check
PLAT767_ALERT_4_C INS Embedded LIST 6 Instruction Should be LIST 4     Please Check
PLAT906_ALERT_3_C Large K Value in the Analysis of Variance ......     19.546 Check
PLAT906_ALERT_3_C Large K Value in the Analysis of Variance ......      4.065 Check
```

Alert level G

```
PLAT720_ALERT_4_G Number of Unusual/Non-Standard Labels .........          2 Note
PLAT883_ALERT_1_G No Info/Value for _atom_sites_solution_primary .     Please Do !
PLAT960_ALERT_3_G Number of Intensities with I  <   - 2*sig(I) ...          8 Check
PLAT965_ALERT_2_G The SHELXL WEIGHT Optimisation has not Converged     Please Check
PLAT967_ALERT_5_G Note: Two-Theta Cutoff Value in Embedded .res ..       55.0 Degree
PLAT978_ALERT_2_G Number C-C Bonds with Positive Residual Density.          0 Info
```

0 **ALERT level A** = Most likely a serious problem - resolve or explain
1 **ALERT level B** = A potentially serious problem, consider carefully
6 **ALERT level C** = Check. Ensure it is not caused by an omission or oversight
6 **ALERT level G** = General information/check it is not something unexpected

2 ALERT type 1 CIF construction/syntax error, inconsistent or missing data
5 ALERT type 2 Indicator that the structure model may be wrong or deficient
3 ALERT type 3 Indicator that the structure quality may be low
2 ALERT type 4 Improvement, methodology, query or suggestion
1 ALERT type 5 Informative message, check

It is advisable to attempt to resolve as many as possible of the alerts in all categories. Often the minor alerts point to easily fixed oversights, errors and omissions in your CIF or refinement strategy, so attention to these fine details can be worthwhile. In order to resolve some of the more serious problems it may be necessary to carry out additional measurements or structure refinements. However, the purpose of your study may justify the reported deviations and the more serious of these should normally be commented upon in the discussion or experimental section of a paper or in the "special_details" fields of the CIF. checkCIF was carefully designed to identify outliers and unusual parameters, but every test has its limitations and alerts that are not important in a particular case may appear. Conversely, the absence of alerts does not guarantee there are no aspects of the results needing attention. It is up to the individual to critically assess their own results and, if necessary, seek expert advice.

Publication of your CIF in IUCr journals

A basic structural check has been run on your CIF. These basic checks will be run on all CIFs submitted for publication in IUCr journals (*Acta Crystallographica*, *Journal of Applied Crystallography*, *Journal of Synchrotron Radiation*); however, if you intend to submit to *Acta Crystallographica Section C* or *E* or *IUCrData*, you should make sure that full publication checks are run on the final version of your CIF prior to submission.

Publication of your CIF in other journals

Please refer to the *Notes for Authors* of the relevant journal for any special instructions relating to CIF submission.

PLATON version of 10/05/2023; check.def file version of 10/05/2023

Datablock mo_s4b_0m - ellipsoid plot

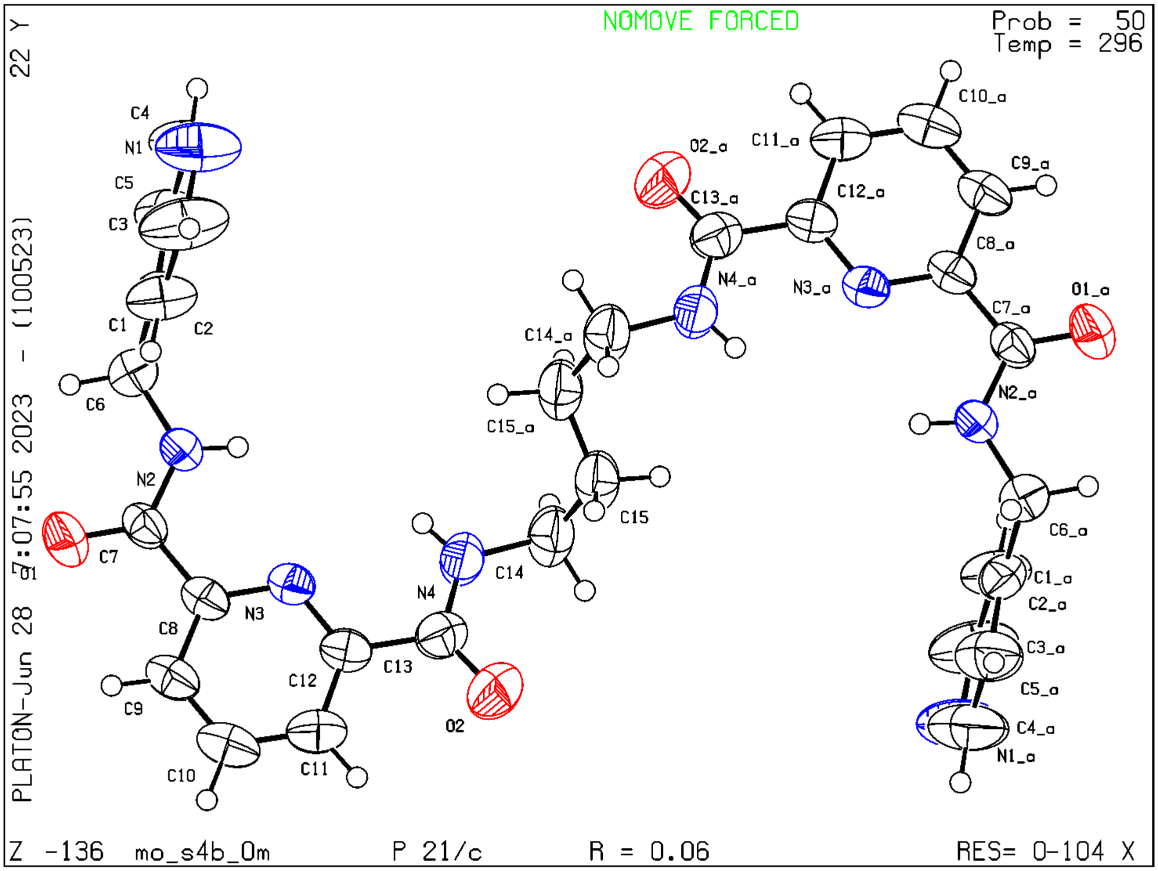

Supplement: Supplementary file 2 [file mmc2.pdf]
